# Marginal fit, internal adaptation, and thermocycling effects on the mechanical and optical properties of graphene CAD-CAM and zirconia crowns for primary molars: An in-vitro study

**DOI:** 10.1016/j.jobcr.2026.101505

**Published:** 2026-08-10

**Authors:** Keshav Rajesh, Jessy Paulraj, Subhabrata Maiti

**Affiliations:** a Department of Pediatric and Preventive Dentistry, Saveetha Dental College and Hospitals, Saveetha Institute of Medical and Technical Sciences, Saveetha University, Chennai, 77, India; b Department of Prosthodontics, Saveetha Dental College and Hospitals, Saveetha Institute of Medical and Technical Sciences, Saveetha University, Chennai, 77, India

**Keywords:** Computer-aided design, Dental marginal adaptation, Dentistry, Graphene, Primary dentition, Surface properties

## Abstract

**Aim:**

To evaluate the marginal fit and internal adaptation of graphene-reinforced CAD-CAM crowns compared with prefabricated zirconia crowns, and to assess the influence of thermocycling on their mechanical and optical properties.

**Materials and methods:**

In this in vitro comparative study, typodont mandibular second primary molars were prepared using a standardized protocol and allocated into two groups: graphene-reinforced CAD-CAM (G-CAM) crowns and prefabricated zirconia crowns. Marginal fit was assessed using a stereomicroscope at 40× magnification, and internal adaptation was evaluated using a silicone replica technique combined with three-dimensional digital analysis. For fracture resistance, crowns were tested under pre- and post-thermocycling conditions using a universal testing machine. Disc specimens were prepared separately for surface roughness, microhardness, and color stability; within each parameter, the same specimens were evaluated before and after thermocycling. Artificial aging was performed using 5000 thermocycles (5°C-55°C). Statistical analysis included Shapiro–Wilk and Levene's tests, independent and paired t-tests, and two-way ANOVA (*P* < 0.05).

**Results:**

G-CAM crowns showed significantly lower marginal discrepancy and internal gap values than zirconia crowns (*P* ≤ 0.002). Fracture resistance was higher in G-CAM crowns before and after thermocycling, with a significant reduction observed in both groups (*P* < 0.05). Surface roughness, microhardness, and color stability were unaffected by thermocycling (*P* > 0.05), with ΔE values within clinically acceptable limits.

**Conclusion:**

Under in vitro conditions, G-CAM crowns demonstrated superior marginal fit, internal adaptation, and fracture resistance compared with zirconia crowns. Thermocycling had minimal effect on surface and optical properties, though clinical studies are needed to confirm long-term performance.

## 1 Introduction

Dental caries remains one of the most prevalent chronic diseases affecting children worldwide and frequently results in extensive destruction of primary teeth. In such situations, restorative treatment plays a crucial role in preserving the remaining tooth structure, restoring masticatory function, maintaining arch integrity, and ensuring proper eruption guidance for permanent successors.^1^ Full-coverage restorations are recommended for primary molars with extensive carious lesions or following pulp therapy procedures such as pulpotomy or pulpectomy.^2^

For several decades, preformed stainless steel crowns (SSCs) have been regarded as the gold standard for restoring severely damaged primary molars because of their excellent durability, longevity, and minimal technique sensitivity.^3^ However, despite their well-documented clinical success, SSCs possess a major limitation related to their metallic and unaesthetic appearance. With increasing parental awareness and demand for esthetic restorations, the use of metal crowns in children has become less desirable, particularly for teeth visible during smiling.

In response to these esthetic concerns, prefabricated zirconia crowns have emerged as a widely accepted alternative for full-coverage restorations in pediatric dentistry. Zirconia crowns offer excellent esthetics, high biocompatibility, and superior flexural strength.^4^ Their monolithic translucent white appearance provides a more natural restoration compared with stainless steel crowns. Nevertheless, prefabricated zirconia crowns present certain inherent limitations. Because they are manufactured in predetermined sizes, achieving optimal adaptation to individual tooth preparations can be challenging. The anatomical mismatch between the prepared tooth and the prefabricated crown may lead to increased marginal discrepancies. Poor marginal adaptation can facilitate microleakage, cement dissolution, plaque accumulation, and secondary caries, ultimately compromising restoration longevity.^5^ Furthermore, zirconia crowns cannot be crimped and require more aggressive tooth preparation to achieve passive seating, which may increase the risk of pulpal exposure in primary teeth. Although zirconia exhibits high strength, its ceramic nature and susceptibility to brittle fracture remain important considerations when used in thin sections under the variable occlusal forces present in pediatric patients.

Recent advancements in digital dentistry have introduced computer-aided design and computer-aided manufacturing (CAD-CAM) technologies that allow the fabrication of customized dental restorations. CAD-CAM systems enable crowns to be designed according to individual tooth morphology and fabricated with high precision, potentially improving marginal fit, internal adaptation, and overall restoration performance.^6^ In pediatric dentistry, CAD-CAM fabricated crowns may offer advantages such as improved aesthetics, enhanced fit, and greater patient and parental acceptance.

The marginal fit and internal adaptation of crown restorations are critical determinants of their long-term clinical success.^7^ Marginal fit refers to the accuracy with which the crown margin adapts to the prepared tooth finish line, whereas internal adaptation describes the uniformity of the internal space between the crown and the tooth surface.^8^ Inadequate marginal adaptation may result in microleakage, bacterial infiltration, cement dissolution, and recurrent caries, ultimately leading to restoration failure. Similarly, poor internal adaptation may create excessive cement thickness, reduce crown retention, and generate unfavorable stress distribution within the restoration. Therefore, achieving optimal marginal fit and internal adaptation is essential for improving the durability and clinical performance of full-coverage restorations.^8^ In addition, restorative materials in the oral cavity are constantly exposed to thermal fluctuations caused by the intake of hot and cold foods and beverages. These temperature variations may induce repeated expansion and contraction of restorative materials, potentially affecting the marginal integrity and internal adaptation of crowns over time. Thermocycling is commonly used in in-vitro studies to simulate these thermal changes and mimic the aging conditions of the oral environment, thereby providing a more clinically relevant evaluation of restorative materials.

With the advancement of digital fabrication methods, the incorporation of nanomaterials into dental polymers has attracted considerable research interest as a strategy to enhance their mechanical performance. Graphene, a two-dimensional carbon nanomaterial consisting of a single layer of carbon atoms arranged in a hexagonal lattice, possesses exceptional properties, including remarkable mechanical strength, superior fracture toughness, excellent wear resistance, and outstanding barrier characteristics.^9^ These unique properties have encouraged its potential application in biomedical and dental materials.

However, a notable concern with the incorporation of graphene into polymethyl methacrylate (PMMA) was its intrinsic dark color, which could adversely affect the esthetics and translucency of tooth-colored restorations. This limitation was particularly relevant in pediatric dentistry, where appearance plays a critical role in treatment acceptance. Nevertheless, this drawback was minimized by using low concentrations of graphene or graphene oxide, which improved mechanical properties without producing clinically perceptible color changes. Furthermore, graphene oxide exhibited a relatively lighter coloration and demonstrated improved dispersion within the PMMA matrix due to its functional groups, thereby enhancing optical blending.^10^ In addition, CAD-CAM fabrication techniques provided better control over material homogeneity and thickness, further reducing the visual impact of the nanofiller and maintaining acceptable esthetic outcomes.^10^

The incorporation of graphene nanoparticles into polymethyl methacrylate (PMMA) represents a promising approach for improving the mechanical properties of polymer-based restorative materials.^11^ Conventional PMMA materials may exhibit limitations such as susceptibility to fracture, water sorption, and relatively low impact resistance. The addition of graphene oxide nanoparticles can significantly enhance the mechanical behavior of PMMA matrices by improving fracture toughness, wear resistance, and structural stability. Graphene oxide contains oxygen-containing functional groups such as hydroxyl and carboxyl groups that facilitate hydrogen bonding and van der Waals interactions with the ester groups of PMMA, thereby improving nanoparticle dispersion and interfacial adhesion within the polymer matrix.^9^

Based on these developments, graphene-reinforced CAD-CAM materials, such as graphene-reinforced PMMA (G-CAM), have recently been introduced as a novel class of restorative materials that combine the advantages of digital fabrication with enhanced mechanical strength. The presence of graphene within the resin matrix may provide improved fracture resistance and durability compared with conventional polymer-based materials while potentially overcoming some of the limitations associated with brittle ceramic materials such as zirconia. Despite these promising developments, limited evidence is currently available regarding the performance of graphene-reinforced CAD-CAM crowns for pediatric restorations. In particular, there is a lack of comparative studies evaluating their marginal fit, internal adaptation, and the influence of thermocycling on their mechanical and optical properties when compared with prefabricated zirconia crowns.

Despite the growing interest in graphene-reinforced CAD-CAM materials, there is a lack of comparative evidence regarding their clinical performance in pediatric dentistry, particularly in comparison with prefabricated zirconia crowns. Most studies have focused on mechanical enhancements, with limited data on clinically relevant parameters such as marginal fit, internal adaptation, and behavior under thermal aging. While zirconia crowns are widely used, their brittleness, lack of customization, and technique sensitivity may affect long-term performance. In contrast, graphene-reinforced CAD-CAM materials are expected to provide improved outcomes due to enhanced fracture resistance, better stress distribution, and the ability to fabricate customized restorations with superior fit. Therefore, comparative evaluation is essential to determine their clinical applicability in pediatric dentistry. The aim of this in vitro study was to evaluate the marginal fit, internal adaptation, and the effect of thermocycling on the mechanical and optical properties of graphene-reinforced CAD-CAM crowns fabricated for primary molars and to compare their performance with prefabricated zirconia crowns. The primary outcome variable of this study was marginal fit, while internal adaptation and mechanical and optical properties were considered secondary outcomes. The null hypothesis of this study was that no significant differences would exist between graphene-reinforced CAD-CAM crowns and prefabricated zirconia crowns with respect to marginal fit, internal adaptation, fracture resistance, hardness, surface roughness, and color stability.

## 2 Materials and methods

### Ethical approval

The study protocol was approved by the Research Ethics Committee of the University Hospital under approval number SRB/SDC/UG-2116/25/PEDO/135.

### 2.1 Study design and sample size determination

This in vitro comparative study evaluated the marginal fit, internal adaptation, fracture resistance, and the influence of thermocycling on the mechanical and optical properties of graphene-reinforced CAD-CAM crowns compared with prefabricated zirconia crowns. Sample size estimation was performed using G*Power software (version 3.1.9.7; Heinrich-Heine University, Düsseldorf, Germany) based on previously reported data. Considering an effect size of 1.51, a two-tailed significance level (α) of 0.05, and a statistical power of 80%, the minimum required sample size was calculated to be 16 specimens (8 per group). To enhance the statistical robustness of the study and to compensate for possible specimen loss during experimental procedures, the sample size was increased, resulting in a base sample size of 40 crown specimens (n = 20 per material group). For the evaluation of thermocycling effects on fracture resistance, each crown group was further subdivided into pre-thermocycling and post-thermocycling conditions (n = 20 per condition), with equal representation of graphene-reinforced CAD-CAM and prefabricated zirconia crowns under each condition. In addition, disc-shaped specimens were fabricated for the evaluation of surface roughness, microhardness, and color stability. For each parameter, 20 zirconia discs and 20 graphene-reinforced CAD-CAM discs were included. The same set of specimens was evaluated before and after thermocycling for each parameter, following a repeated-measures design.

### 2.2 Materials used and sample allocation

The graphene-reinforced CAD-CAM material used in this study was a commercially available graphene-modified polymethyl methacrylate (PMMA) disc (G-CAM) (Graphenano Dental, Valencia, Spain; Lot No.: as per manufacturer's batch used in the laboratory). The material consists of a PMMA-based resin matrix reinforced with graphene nanoparticles, designed for CAD-CAM milling applications. The exact graphene concentration is proprietary to the manufacturer. All specimens were fabricated from the same batch to ensure standardization. All other materials and equipment used in the study are summarized in Table 1.

**Table 1:**
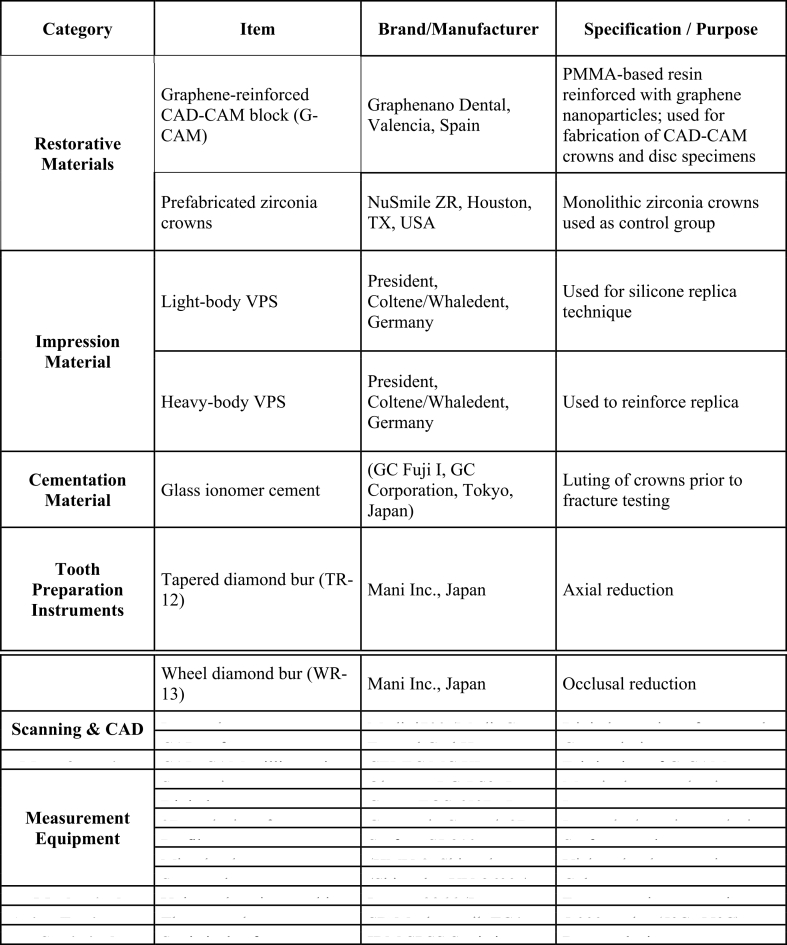
Materials, equipment, and software used in the Study.

**Fig. 1:**
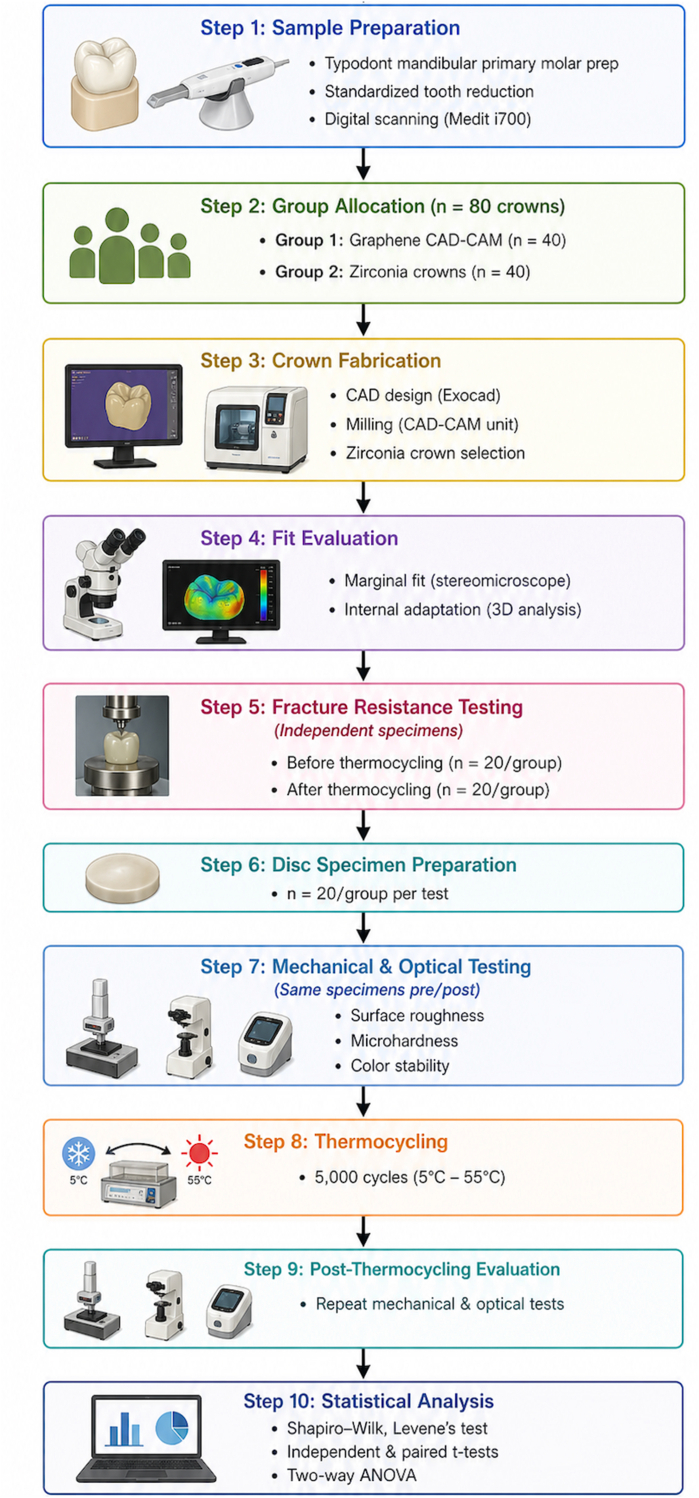
Schematic overview of experimental workflow and allocation.

The specimens were randomly assigned into two experimental groups using computer-generated randomization (Research Randomizer software). Allocation was performed by an independent investigator to minimize selection bias. Group 1: Graphene-reinforced CAD-CAM crowns (G-CAM) (Graphenano Dental, Valencia, Spain) (n = 40), subdivided into pre-thermocycling (n = 20) and post-thermocycling (n = 20) subgroups. Group 2: Prefabricated zirconia crowns (NuSmile ZR, NuSmile, Houston, TX, USA) (n = 40), subdivided into pre-thermocycling (n = 20) and post-thermocycling (n = 20) subgroups. For the evaluation of surface roughness, microhardness, and color stability, disc specimens were prepared separately for each parameter. For each parameter, 20 zirconia discs and 20 graphene-reinforced CAD-CAM discs were used (n = 20 per material). The same specimens were evaluated before and after thermocycling for each parameter. In total, this resulted in 60 zirconia discs and 60 graphene-reinforced CAD-CAM discs across all three parameters. The overall experimental workflow and specimen allocation are illustrated in Fig. 1.

### 2.3 Standardized tooth preparation

**Fig. 2:**
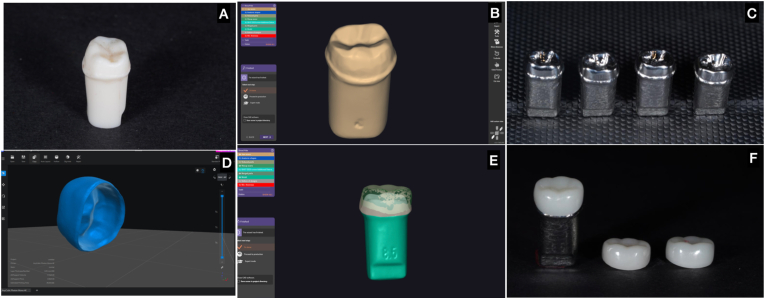
Workflow for crown design and specimen preparation (A) Prepared typodont tooth following standardized crown preparation. (B) Digital scan and 3D reconstruction of the prepared tooth. (C) Fabricated metal dies used as standardized substrates for crown evaluation. (D) Digital design of zirconia crown used as a reference for standardization. (E) Virtual adaptation of the designed crown on the prepared tooth model during the CAD workflow. (F) Final crowns used in the study, including the prefabricated zirconia crown and milled graphene-reinforced CAD-CAM crowns prior to testing.

All preparations were performed by a single calibrated operator using a high-speed contra-angle handpiece under continuous water irrigation. Prior to specimen preparation, the operator was calibrated through repeated preparation procedures on typodont teeth to ensure consistency and reproducibility of the preparation dimensions. To assess intra-examiner reliability, marginal gap measurements were repeated for five randomly selected specimens after a one-week interval, and the consistency of measurements was verified. Axial reduction was performed using a blue-coded tapered diamond bur with a round end (Mani TR-12, Mani Inc., Japan) to achieve 0.7–1 mm circumferential axial reduction, producing a chamfer finish line. Occlusal reduction of approximately 1.5 mm was achieved using a wheel diamond bur (Mani WR-13, Mani Inc., Japan) to provide sufficient occlusal clearance (Fig. 2A). Following preparation, the typodont tooth was digitally scanned using an intraoral scanner (Medit i700, Medit Corp., Seoul, Republic of Korea)) to generate a high-resolution digital model (Fig. 2B).

### 2.4 Crown design and fabrication

Following standardized tooth preparation on the typodont model, the prepared tooth was used to fabricate a metal die to ensure dimensional stability and reproducibility during crown fabrication and evaluation. An impression of the prepared typodont tooth was obtained and used to produce a metal die using a cobalt–chromium alloy through conventional casting procedures (Fig. 2C). The metal die served as a standardized master model for crown adaptation and testing, minimizing dimensional variations associated with repeated use of the typodont model. After tooth preparation, an appropriately sized prefabricated zirconia crown (NuSmile ZR) was selected according to the manufacturer's recommendations to achieve optimal clinical fit. To ensure methodological standardization and minimize variations in crown morphology during the fabrication of graphene-reinforced CAD-CAM (G-CAM) crowns, the selected zirconia crown was used as a reference during the digital design process (Fig. 2D). The external morphology and dimensions of the prefabricated crown were digitally replicated to maintain consistent crown thickness, contour, and occlusal morphology across all specimens. The prepared typodont tooth was digitally scanned, and the scan data were imported into Exocad CAD software (Exocad GmbH, Darmstadt, Germany) for crown design. For the G-CAM group, crowns were designed with a standardized thickness of approximately 1 mm across the occlusal, buccal, lingual, and proximal surfaces (Fig. 2E). The finalized designs were exported as STL files and milled from graphene-reinforced CAD-CAM blocks using a computer-aided milling system. The fabricated crowns were then finished and polished according to the manufacturer's instructions (Fig. 2F). For the zirconia group, prefabricated zirconia crowns (NuSmile ZR) were selected from the manufacturer's kit and adapted to the prepared typodont tooth and corresponding metal die to achieve complete seating. Minor adjustments were performed when necessary to ensure proper adaptation and standardized fit across all specimens.

### 2.5 Marginal fit evaluation

**Fig. 3:**
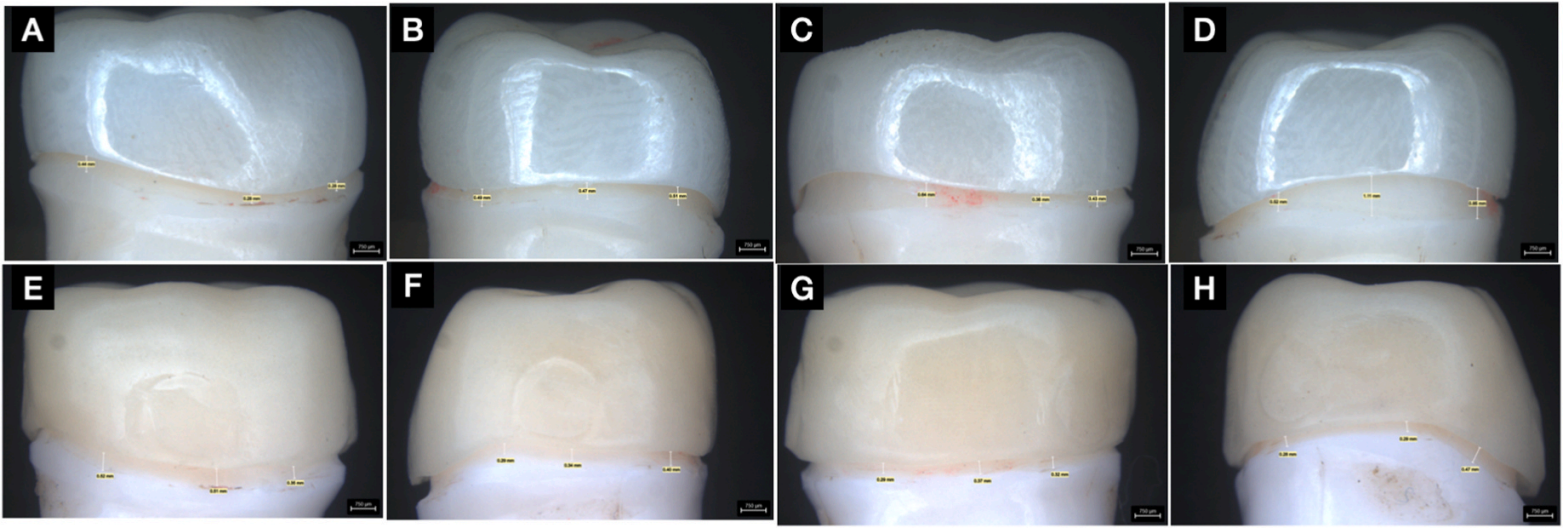
Stereomicroscopic evaluation of marginal fit of prefabricated zirconia and graphene-reinforced CAD-CAM crowns. Representative stereomicroscopic images showing marginal gap measurements at four tooth surfaces. The upper row (A–D) represents prefabricated zirconia crowns, while the lower row (E− H) represents graphene-reinforced CAD-CAM (G-CAM) crowns. Marginal gaps were measured at the buccal (A, E), distal (B, F), lingual (C, G), and mesial (D, H)surfaces using a stereomicroscope.

Marginal fit was evaluated using a stereomicroscope (LG-PS2, Olympus, Japan) equipped with a digital camera system (Canon EOS 650D, Japan) at 40× magnification. Each crown was seated on the prepared typodont tooth under quantified seating force (N). Measurements were obtained at standardized regions, including the buccal, lingual, mesial, and distal surfaces, and the mean value of multiple measurements per specimen was calculated. Digital images were captured and analyzed using calibrated image analysis software, and marginal gap values were initially recorded in millimeters (mm) and subsequently converted to micrometers (μm) for statistical analysis for both zirconia crowns (Fig. 3A–D) and G-CAM crowns (Fig. 3E–H). Each measurement was repeated three times and the mean value was calculated.

### 2.6 Internal adaptation evaluation (silicone replica technique and 3D digital analysis)

**Fig. 4:**
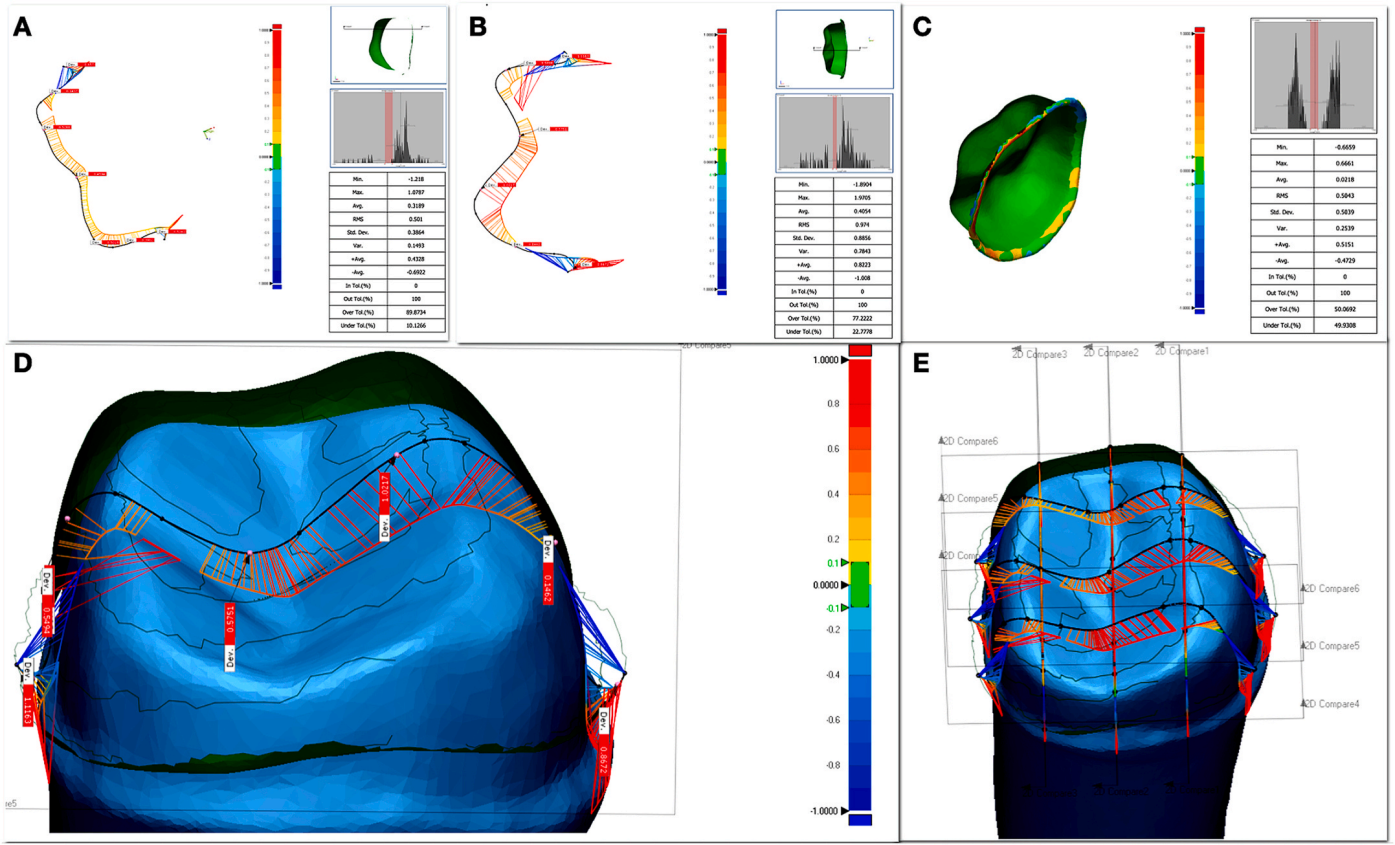
Internal adaptation analysis of G-CAM Crown using Geomagic software. (A–C) Cross-sectional and 3D deviation maps obtained by superimposing the crown intaglio surface with the prepared tooth. The color scale indicates internal discrepancy (green = optimal fit; yellow–red = increased gap; blue = reduced space/contact).(D) Sectional view showing internal gap measurements along the occlusal and axial surfaces.(E) Overall 3D deviation map illustrating the distribution of internal adaptation across the crown–tooth interface.

**Fig. 5:**
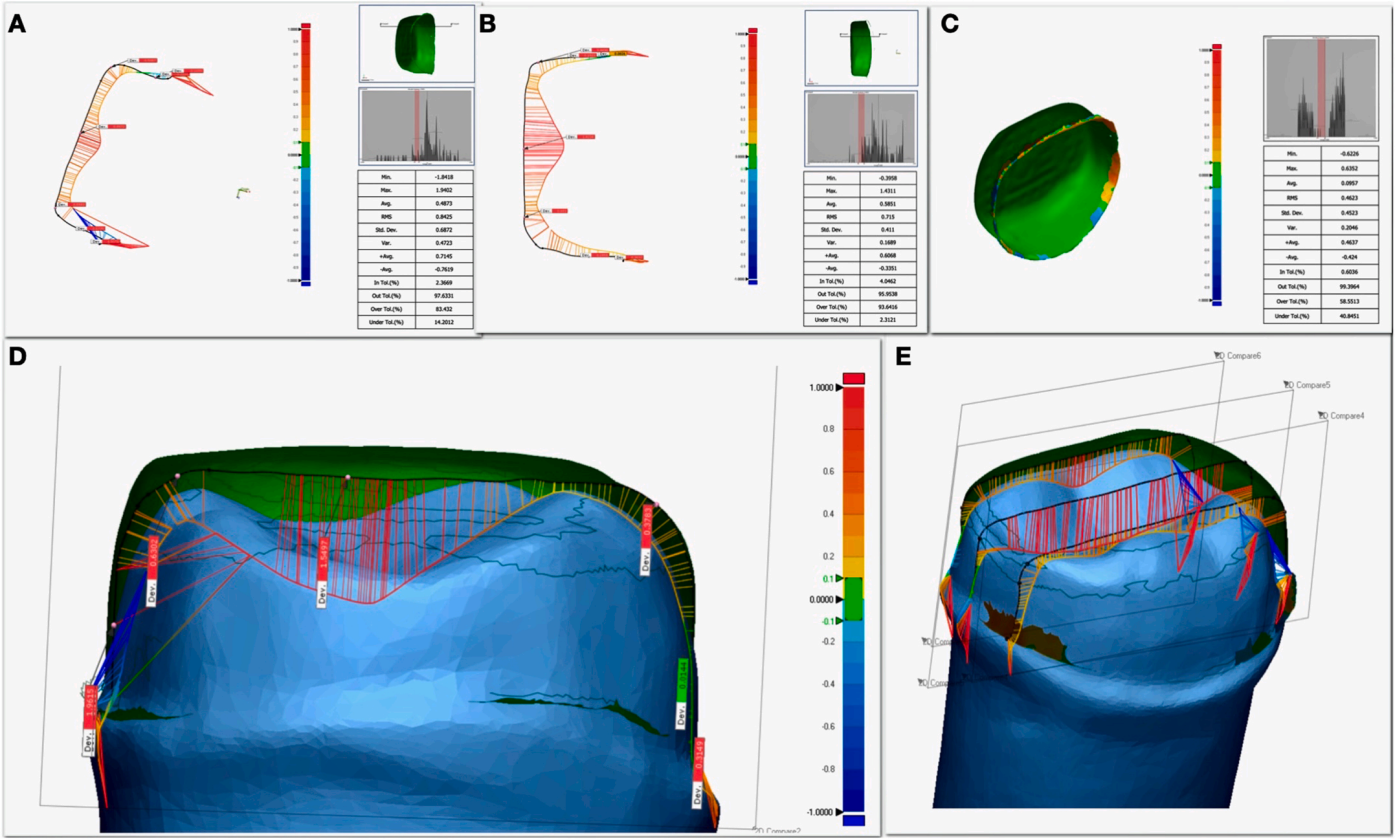
Digital assessment of crown internal adaptation of zirconia crown using Geomagic software. (A–C) Cross-sectional and three-dimensional color-coded deviation maps generated after superimposition of the crown intaglio surface with the prepared tooth. The color spectrum represents the magnitude of internal discrepancy (green = optimal adaptation; yellow–red = increased internal gap; blue = negative deviation or contact).(D) Sectional view demonstrating internal gap measurements at the occlusal regions.(E) Comprehensive 3D deviation map illustrating the distribution of internal adaptation across the crown–tooth interface.

Internal adaptation analysis was performed using Geomagic Control software (3D Systems, USA). The STL files of the scanned silicone replicas and reference models were imported into the software for analysis. The alignment between the scanned replica and reference model was achieved using a best-fit alignment algorithm. A tolerance range of ±10 μm was set for deviation analysis. The software calibration was verified prior to analysis using the manufacturer's standard calibration protocol to ensure measurement accuracy and reproducibility. Internal adaptation between the crown and the prepared tooth was assessed using the silicone replica technique combined with three-dimensional digital analysis. The intaglio surface of each crown was filled with light-body vinyl polysiloxane impression material (President, Coltene/Whaledent, Germany) to simulate the luting cement layer. Each crown was then seated on the prepared typodont tooth under standardized finger pressure for approximately 3 min to ensure complete seating and uniform distribution of the material. After polymerization, the crown was carefully removed, leaving a thin silicone layer that represented the internal cement space. This layer was reinforced with heavy-body vinyl polysiloxane to produce a stable silicone replica of the internal gap. The replicas were subsequently scanned from all surfaces using a high-resolution laboratory scanner to generate three-dimensional digital datasets. The scanned files were exported in STL format and imported into Geomagic Control software (3D Systems, USA) for digital analysis. Using the software's best-fit alignment algorithm, the replica dataset was superimposed onto the reference digital model of the prepared tooth. Three-dimensional gap analysis was then performed to determine the internal discrepancy between the crown and the tooth preparation. Measurements were obtained from standardized regions, including the occlusal surface, axial walls, and marginal area, for G-CAM crowns (Fig. 4A–E) and zirconia crowns (Fig. 5A–E). Internal adaptation values were expressed in micrometers (μm), and the mean values were used for statistical analysis.

### 2.7 Specimen preparation

Disc-shaped specimens measuring 5 mm in diameter and 7 mm in thickness were fabricated from graphene-reinforced CAD-CAM material and zirconia for the evaluation of mechanical and optical properties before and after thermocycling. The specimens were digitally designed using CAD software and subsequently milled using a CAD-CAM milling unit according to the manufacturer's instructions.

### 2.8 Mechanical properties

#### 2.8.1 Fracture resistance testing

**Fig. 6:**
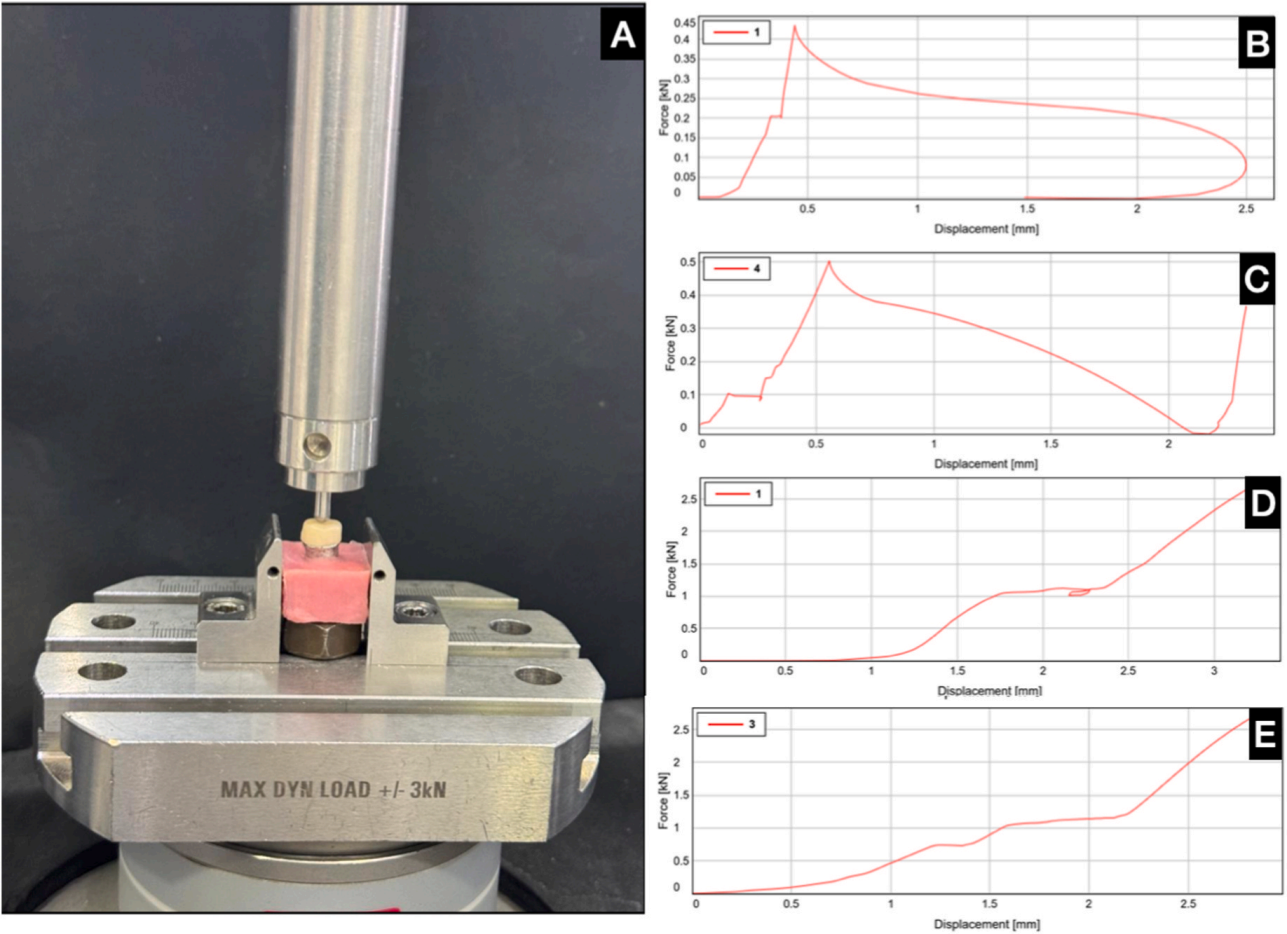
Compressive Load Testing of Crown Specimens to Assess Fracture Resistance (A) Experimental setup for compressive load testing using a universal testing machine, where the crown specimen was positioned on the die and subjected to vertical loading until failure. (B–C) Representative force–displacement curves obtained for prefabricated zirconia crowns during compressive loading. (D–E) Representative force–displacement curves recorded for graphene-reinforced CAD-CAM crowns, illustrating the load response until fracture.

Prior to fracture resistance testing, all crowns were cemented onto the prepared tooth using glass ionomer luting cement according to the manufacturer's instructions. A thin layer of cement was applied to the intaglio surface of the crown, and the restoration was seated under standardized finger pressure. Excess cement was removed after initial setting. All specimens were stored in distilled water at 37°C for 24 h before mechanical testing. To ensure adequate support during mechanical testing, metal dies made from chromium–cobalt (Cr–Co) alloy were fabricated using CAD–CAM technology based on the digital scan of the prepared tooth. Each crown was placed on its corresponding metal die, and proper seating was verified using a fit-checking material (GC Fit Checker, GC Corporation, Tokyo, Japan). The crown–die assemblies were then embedded in auto-polymerizing acrylic resin to achieve standardized positioning during testing. Fracture resistance was evaluated using a universal testing machine Instron 3366 (Instron Corp., Norwood, MA, USA). A compressive load was applied through a stainless-steel loading tip oriented at 135° to the long axis of the tooth to simulate functional occlusal forces (Fig. 6A). The load was delivered at a crosshead speed of 0.5 mm/min until catastrophic failure of the crown occurred. All tests were performed under controlled laboratory conditions at 23 ± 1°C temperature and 50 ± 5% relative humidity. The maximum load at fracture was recorded in Newtons (N) (Fig. 6B–E).

#### 2.8.2 Surface roughness

**Fig. 7:**
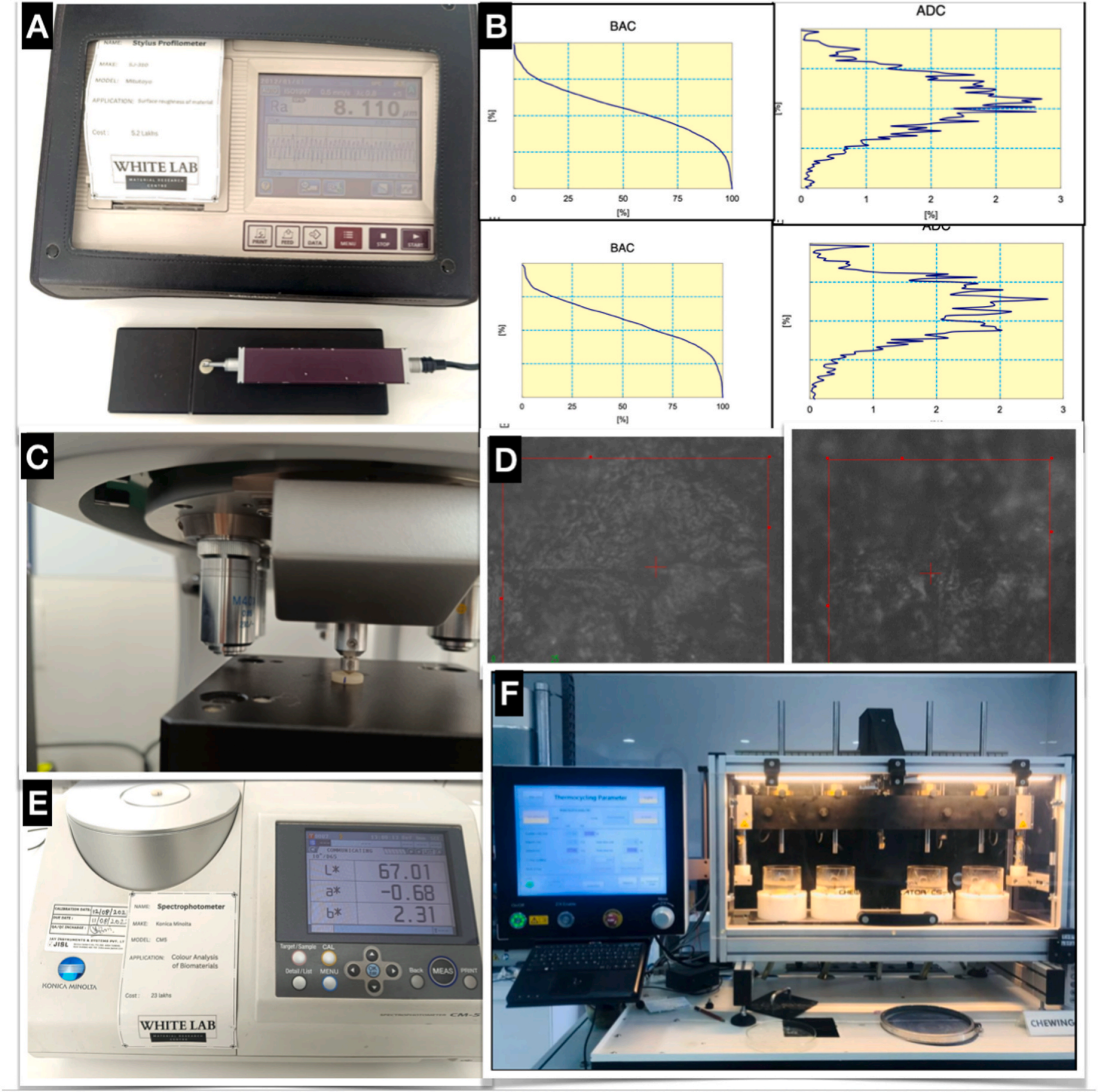
Evaluation of mechanical and optical properties of materials. (A–B) Surface roughness assessment using a stylus profilometer and corresponding roughness profile graphs. (C–D) Microhardness evaluation performed using a Vickers microhardness tester and representative microscopic indentation images. (E) Color stability analysis carried out using a spectrophotometer based on CIE L*a*b* color parameters. (F) Thermocycling procedure performed to simulate oral thermal aging conditions prior to testing.

Surface roughness (Ra) was measured using a contact profilometer (Surftest SJ-210, Mitutoyo Corp., Japan) with a cut-off length of 0.8 mm and a tracing speed of 0.5 mm/s. Three readings were obtained from different areas of each specimen and the mean value was recorded (Fig. 7A-B).

#### 2.8.3 Surface hardness

Surface hardness was determined using a Vickers microhardness tester (HMV-2, Shimadzu Corp., Kyoto, Japan) with a load of 200 g applied for 15 s. Three indentations were made on each specimen surface and the mean hardness value was calculated (Fig. 7C-D).

### 2.9 Optical properties

#### 2.9.1 Color stability

Color measurements of the specimens were obtained using a spectrophotometer (Shimadzu UV-2600/UV-1800, Shimadzu Corporation, Kyoto, Japan) under standardized illumination conditions. The device was calibrated prior to each measurement using a standard white calibration tile. Color values were recorded according to the CIELAB color system (L, a, b*), and the overall color difference (ΔE) before and after thermocycling was calculated (Fig. 7E).

### 2.10 Thermocycling procedure

Artificial aging was performed using a thermocycling device (Thermocycler TC4, SD Mechatronik GmbH, Germany). Specimens were subjected to 5000 thermocycles between 5°C and 55°C, with a dwell time of 30 s in each bath and a transfer time of 10 s, simulating approximately six months of intraoral service (Fig. 7F). After thermocycling, the specimens were re-evaluated for Fracture resistance, surface hardness, surface roughness, and color stability.

### 2.11 Intra-examiner reliability

Intra-examiner reliability was assessed using the intraclass correlation coefficient (ICC) based on repeated measurements of 10% of the specimens after a one-week interval. The ICC value was 0.92, indicating excellent reliability and consistency of measurements.

### 2.12 Statistical analysis

Statistical analysis was performed using IBM SPSS Statistics software (IBM Corp., Armonk, NY, USA). Data were expressed as mean ± standard deviation (SD). The normality of data distribution was assessed using the Shapiro–Wilk test, and homogeneity of variances was evaluated using Levene's test. For marginal fit and internal adaptation, comparisons between graphene CAD-CAM and prefabricated zirconia crowns were performed using independent samples t-tests. For fracture resistance, separate crown specimens were used for testing before and after thermocycling due to the destructive nature of the test. Therefore, comparisons within each material and between materials were performed using independent samples t-tests. For disc specimens (surface roughness, microhardness, and color stability), independent specimen sets were used for each parameter; however, within each parameter, the same specimens were evaluated before and after thermocycling. Accordingly, paired samples t-tests were applied for within-group comparisons. Additionally, a two-way analysis of variance (ANOVA) was performed to evaluate the main effects of material type and thermocycling, as well as their interaction, on fracture resistance. Mean differences along with 95% confidence intervals (CI) were calculated for all relevant comparisons. Given the evaluation of multiple outcome variables, the potential for type I error inflation was considered. However, as marginal fit was defined as the primary outcome, no adjustment was applied for this parameter. Secondary outcomes were interpreted with consideration of multiple comparisons using Bonferroni correction. The assumptions required for parametric testing, including normal distribution and homogeneity of variance, were verified prior to analysis. The level of statistical significance was set at *P* < 0.05.

## 3 Results

All variables were normally distributed (Shapiro–Wilk test, *P* > 0.05) and demonstrated homogeneity of variance (Levene's test, *P* > 0.05), confirming the suitability of parametric statistical analysis (Table 2). For fracture resistance, independent crown specimens were used for pre- and post-thermocycling conditions due to the destructive nature of the test. For disc specimens (surface roughness, microhardness, and color stability), separate groups of specimens were used for each parameter; however, within each parameter, the same specimens were evaluated before and after thermocycling.

**Table 2:** Assessment of normality and homogeneity of variance using Shapiro–Wilk and Levene's tests.

Parameter	Group	Shapiro–Wilk (*P*-value)	Levene's Test (*P*-value)	Normality	Homogeneity of Variance
Marginal Fit	G-CAM	0.214	0.327	Normal	Equal variance
	Zirconia	0.189			
Internal Adaptation	G-CAM	0.176	0.291		
	Zirconia	0.203			
Fracture Resistance	G-CAM	0.221	0.348		
	Zirconia	0.195			
Surface Roughness	G-CAM	0.265	0.401		
	Zirconia	0.238			
Microhardness	G-CAM	0.198	0.376		
	Zirconia	0.211			
Color Stability	G-CAM	0.229	0.354		
	Zirconia	0.217			

*P* > 0.05 indicates normal distribution (Shapiro–Wilk test) and equal variances (Levene's test).

### 3.1 Marginal fit

The mean marginal fit values demonstrated a statistically significant difference between the two crown systems. Graphene CAD-CAM crowns exhibited a significantly lower marginal discrepancy (68.4 ± 5.2 μm; 95% CI: 65.9–70.9) compared with prefabricated zirconia crowns (92.7 ± 6.8 μm; 95% CI: 89.4–96.0). The mean difference between groups was statistically significant, with a mean difference of −24.3 μm (95% CI: −28.5 to −20.1). The independent samples *t*-test revealed a statistically significant difference in marginal fit between the groups (p = 0.002) (Table 3). The magnitude of the difference between groups was large, as indicated by effect size analysis (Cohen's d > 1.0).

**Table 3:** Comparison of Marginal Fit and Internal Adaptation Between Graphene CAD-CAM (G-CAM) and Zirconia Crowns Using Independent Samples *t*-test (Mean ± SD, Mean Difference, and 95% Confidence Intervals).

Parameter	Group	n	Mean ± SD	Mean Difference (95% CI)	*P*-value
Marginal Fit	G-CAM crown	20	68.4 ± 5.2	−24.3 (−28.5 to −20.1)	0.002*
	Zirconia Crown	20	92.7 ± 6.8		
Internal Adaptation	G-CAM Crown	20	85.6 ± 7.1	−26.7 (−31.2 to −22.2)	0.0008*
	Zirconia Crown	20	112.3 ± 8.5		

Statistically significant difference (*P* < 0.05).

CI: Confidence interval; SD: Standard deviation.

Between-group comparisons performed using independent *t*-test.

### 3.2 Internal adaptation

Similarly, a statistically significant difference was observed in the three-dimensional internal adaptation between the two groups. Graphene CAD-CAM crowns demonstrated significantly lower internal gap values (85.6 ± 7.1 μm; 95% CI: 82.1–89.0) compared with prefabricated zirconia crowns (112.3 ± 8.5 μm; 95% CI: 108.2–116.4) (Table 3). The independent *t*-test showed that the mean difference in internal adaptation between groups was −26.7 μm (95% CI: −31.2 to −22.2). The difference between the groups was statistically significant (*P* < 0.001).

#### 3.2.1 Evaluation of mechanical properties before and after thermocycling

##### 3.2.1.1 Fracture resistance

Fracture resistance of the specimens was evaluated using a universal testing machine, and the fracture load was recorded in Newtons (N). Before thermocycling, graphene CAD-CAM (G-CAM) specimens demonstrated a mean fracture resistance of 1785 ± 120 N, whereas zirconia crowns showed a mean value of 1420 ± 105 N. After thermocycling, fracture resistance decreased in both groups, with GCAM specimens recording 1710 ± 115 N and zirconia crowns 1315 ± 98 N (Table 4). Independent samples t-tests showed a statistically significant reduction in fracture resistance after thermocycling in both G-CAM (*P* = 0.041) and zirconia crowns (*P* = 0.032). Two-way ANOVA revealed a significant effect of material (F(1,76) = 18.52, *P* = 0.001) and thermocycling (F(1,76) = 6.34, *P* = 0.016) on fracture resistance. No statistically significant interaction effect was observed (F(1,76) = 1.11, *P* = 0.298) (Table 5).

**Table 4:** Mechanical and optical properties of graphene CAD-CAM(G-CAM) and zirconia materials before and after thermocycling (independent and paired comparisons).

Parameter	Material	Before thermocycling (Mean ± SD)	After thermocycling (Mean ± SD)	Mean Change (95% CI)	*P*-value
Fracture Resistance	G-CAM	1785 ± 120	1710 ± 115	75 (10 to 140)	0.041*
	Zirconia	1420 ± 105	1315 ± 98	105 (20 to 190)	0.032*
Surface Roughness	G-CAM	0.32 ± 0.04	0.33 ± 0.05	0.01 (−0.01 to 0.03)	0.39
	Zirconia	0.34 ± 0.05	0.35 ± 0.06	0.01 (−0.02 to 0.03)	0.41
Microhardness	G-CAM	845 ± 26	838 ± 29	−7 (−18 to 4)	0.48
	Zirconia	852 ± 27	846 ± 30	−6 (−20 to 8)	0.45
Color Stability (ΔE)	G-CAM	1.82 ± 0.31	2.08 ± 0.42	0.26 (−0.10 to 0.62)	0.37
	Zirconia	1.90 ± 0.35	2.15 ± 0.44	0.25 (−0.12 to 0.62)	0.40

Fracture resistance comparisons were performed using independent samples t-tests (different specimens used before and after thermocycling). Surface roughness, microhardness, and color stability were analyzed using paired samples t-tests (same specimens evaluated before and after thermocycling).

Statistically significant difference (*P* < 0.05).

CI: Confidence interval; SD: Standard deviation.

Within-group comparisons performed using paired *t*-test.

**Table 5:** Two-way ANOVA evaluating the effects of material and thermocycling on fracture resistance (N).

Source of Variation	df	F-value	*P*-value	Partial η^2^*	Interpretation
Material (G-CAM vs Zirconia)	1, 76	18.52	0.001*	≈0.20	Significant
Thermocycling (Before vs After)	1, 76	6.34	0.016*	≈0.08	Significant
Material × Thermocycling	1, 76	1.11	0.298	≈0.018	Not significant

*P* < 0.05 indicates statistical significance.

Partial η^2^ represents effect size (0.01 = small, 0.08 = moderate, 0.20 = large).

##### 3.2.1.2 Surface roughness

Surface roughness (Ra) of the disc specimens was measured using a surface profilometer before and after thermocycling. G-CAM specimens showed a mean surface roughness of 0.32 ± 0.04 μm before thermocycling and 0.33 ± 0.05 μm after thermocycling. Zirconia specimens demonstrated mean values of 0.34 ± 0.05 μm before thermocycling and 0.35 ± 0.06 μm after thermocycling (Table 4). No statistically significant differences were detected between pre- and post-thermocycling measurements (*P* = 0.39 for G-CAM; *P* = 0.41 for zirconia).

##### 3.2.1.3 Surface hardness

Microhardness was evaluated using a Vickers microhardness tester. G-CAM specimens exhibited mean hardness values of 845 ± 26 VHN before thermocycling and 838 ± 29 VHN after thermocycling. Zirconia specimens showed mean values of 852 ± 27 VHN before thermocycling and 846 ± 30 VHN after thermocycling (Table 4). Statistical analysis indicated no significant differences following thermocycling (*P* = 0.48 for G-CAM; *P* = 0.45 for zirconia).

### 3.3 Evaluation of optical properties before and after thermocycling

#### 3.3.1 Color stability

Color stability was assessed by measuring the color difference values (ΔE) (Table 4). G-CAM specimens showed a mean ΔE value of 1.82 ± 0.31 before thermocycling and 2.08 ± 0.42 after thermocycling. Zirconia specimens demonstrated mean ΔE values of 1.90 ± 0.35 before thermocycling and 2.15 ± 0.44 after thermocycling. No statistically significant differences were identified (*P* = 0.37 for G-CAM; *P* = 0.40 for zirconia).

## 4 Discussion

Restoration of pulpally treated primary molars remains a significant clinical challenge. While prefabricated metal crowns have long been regarded as the gold standard for their durability and predictable clinical performance,^12^ increasing parental demand for esthetic, tooth-colored alternatives has led to widespread adoption of zirconia crowns in pediatric dentistry. Prefabricated zirconia crowns (ZRCs) are considered superior to metal counterparts in terms of esthetics and biocompatibility, yet their predetermined sizes and geometries can limit precise adaptation to individual tooth morphology, often necessitating additional preparation or adjustment.^13^ In contrast, CAD/CAM technologies facilitate the fabrication of customized crowns that conform precisely to the prepared tooth architecture, potentially improving adaptation and conserving tooth structure. Numerous investigations support this, reporting that digitally designed and milled crowns often demonstrate improved precision and fit compared with stock prefabricated designs.^14^^,^^15^

In the present in-vitro study, graphene-reinforced CAD/CAM (G-CAM) crowns demonstrated significantly better performance than prefabricated zirconia crowns in terms of marginal fit, internal adaptation, and fracture resistance. The G-CAM group exhibited considerably smaller mean marginal gaps (∼68 μm), whereas zirconia crowns presented wider discrepancies (∼93 μm). Both values fall within the generally accepted clinical threshold for marginal gaps (≤100–150 μm) reported in restorative dentistry, but the marked improvement seen with GCAM crowns underscores the precision advantages inherent to a digital workflow. High marginal adaptation is clinically critical, as a tighter marginal seal minimizes cement exposure, reduces microleakage, and may decrease the risk of recurrent caries and gingival inflammation over the restoration's lifespan.^16^ The superior marginal fit observed in G-CAM crowns aligns with findings from studies comparing digital CAD/CAM restorations with stock prosthesis systems, which report enhanced conformity and precision when crowns are designed and milled to match individual dental preparations.^14^ In contrast, Al-Haj et al.,^16^ reported that prefabricated zirconia crowns exhibited similar marginal discrepancies when evaluated via sectioning techniques, suggesting that methodological differences may influence reported outcomes.

Internal adaptation followed a similar pattern, with G-CAM crowns showing smaller internal gaps than zirconia crowns. Reduced internal discrepancies are desirable as they contribute to uniform cement thickness, better stress distribution, and improved retention. The superior internal adaptation of graphene-reinforced CAD-CAM crowns may be attributed to their favorable machinability and material behavior during milling. The graphene–PMMA composite is less brittle and more homogeneous than zirconia, allowing smoother CAD-CAM milling with reduced micro-chipping and surface irregularities. In contrast, zirconia's inherent brittleness and high hardness may lead to milling-induced micro-defects, affecting intaglio surface accuracy. Additionally, graphene reinforcement improves stress distribution within the polymer matrix, preserving surface integrity during fabrication.^17^ These factors collectively result in a smoother internal surface and better adaptation to the tooth structure, thereby reducing internal gap values compared with zirconia crowns. Previous literature has demonstrated that excessive internal space can compromise mechanical stability and increase the potential for cement dissolution under functional loading.^18^ Supporting this, El-Farag et al., observed significantly lower internal gaps in custom-designed CAD/CAM crowns compared with stock ceramic crowns, reinforcing the advantage of individualized digital design.^19^ Conversely, some studies have noted minimal differences between CAD/CAM and prefabricated crowns with regard to internal adaptation, depending on the materials and fabrication protocols employed.^20^ The use of an integrated silicone replica technique coupled with 3D digital analysis in this study provided a comprehensive evaluation of internal adaptation across occlusal, axial, and marginal regions.

A notable difference between the two materials was observed in fracture resistance. G-CAM crowns showed significantly higher fracture resistance both before and after thermocycling compared with zirconia crowns. Supporting evidence indicates that CAD/CAM resin-based crown systems often exhibit higher fracture resistance than prefabricated zirconia crowns after artificial aging, likely reflecting material architecture and reinforcement strategies.^21^ Zirconia, though inherently hard, is a brittle ceramic susceptible to flaw initiation and propagation when subjected to occlusal forces, particularly when minor surface adjustments or cementation procedures introduce microstructural defects. In contrast, Hajaj T et al.,reported that some zirconia CAD/CAM crowns still demonstrated high fracture resistance compared with certain ceramics, highlighting that fracture behavior can be material-specific within the CAD/CAM systems.^22^ In pediatric dentistry, where masticatory forces can fluctuate widely and parafunctional habits may be present, a higher margin of mechanical safety is desirable. Owais et al., stated that bite force studies show that mean occlusal forces in primary dentition often remain below 300 N, even in later developmental stages, yet higher forces can occur and challenge restoration integrity.^23^ Both materials tested exceeded these functional requirements, but the higher fracture thresholds recorded for G-CAM crowns suggest that they may offer enhanced durability and longevity, especially in demanding clinical scenarios.

Thermocycling, used to simulate intraoral aging, had minimal influence on the mechanical and surface properties of both materials. Slight reductions in fracture resistance were observed; however, these changes were not statistically significant in terms of interaction effects^.^ Additionally, surface roughness measurements before and after thermocycling remained in the range considered clinically acceptable (Ra <0.5 μm), with no statistically significant differences between groups or time points. This finding is corroborated by studies assessing artificial aging's impact on CAD/CAM restorations, which generally report only modest alterations in performance metrics over simulated time periods.^24^^,^^25^ Bozogullari and Temizci found that CAD/CAM resin-based and ceramic crowns exhibited no significant change in surface roughness after thermocycling, consistent with the present results. Hardness values, measured using Vickers microhardness testing, were also comparable between G-CAM and zirconia crowns as values remained stable across both groups before and after thermocycling and were within reported ranges for dental restorative materials.^26^^,^^27^ Color stability, evaluated via ΔE values using the CIELAB system, remained within clinically acceptable limits for both materials, with no significant changes after thermocycling. Previous studies have suggested that ΔE values below the threshold of ∼3.3 are perceptually acceptable and unlikely to raise esthetic concerns, aligning with the present results.^28^^,^^29^ In contrast, studies that utilized prolonged staining solutions showed more pronounced color shifts, indicating that factors beyond thermal aging may influence long-term esthetics.^30^

Contrasting studies exist regarding adaptation and mechanical performance of dental crown materials. For instance, some investigations have reported smaller marginal gaps in sectioned zirconia specimens compared with impressions or replica methods, likely due to methodological differences rather than material superiority.^16^ Others have noted that sintering shrinkage and milling inaccuracies can negatively affect fit in some CAD-CAM systems, emphasizing the importance of accurate calibration and milling strategies to optimize outcomes. Similarly, while traditional ceramics exhibit high hardness and esthetic translucency, their fracture toughness may lag behind reinforced composite systems under complex loading conditions.^31^, ^32^, ^33^ The superior marginal fit and internal adaptation observed for G-CAM crowns in the present study may be attributed to the precision of the CAD-CAM digital workflow, which enables customized restorations that better conform to tooth morphology. Similar findings were reported by Bhartiya et al., who observed acceptable marginal discrepancy and favorable internal adaptation with CAD-CAM fabricated G-CAM crowns.^17^ Furthermore, the significantly higher fracture resistance recorded in the present study is consistent with El Hayek et al., who demonstrated greater fracture strength for custom-fabricated crowns compared with prefabricated zirconia crowns in primary molars.^34^ However, some studies report that prefabricated zirconia crowns also exhibit clinically acceptable marginal adaptation and mechanical strength, likely due to the inherent properties of zirconia ceramics.

The statistical approach adopted in the present study further strengthens the reliability of the findings. Appropriate statistical tests were selected based on the study design, including independent comparisons for fracture resistance due to the use of separate specimens and paired analyses for repeated measurements within the same samples. Additionally, the use of two-way analysis of variance enabled simultaneous evaluation of the effects of material type and thermocycling, as well as their interaction. This comprehensive analytical framework enhances the robustness of the results and supports the validity of the observed differences between the groups.

The current investigation revealed statistically significant differences between graphene-reinforced CAD-CAM crowns and prefabricated zirconia crowns in terms of marginal fit, internal adaptation, and fracture resistance under in vitro conditions. Graphene CAD-CAM crowns exhibited lower marginal and internal discrepancies and higher fracture resistance compared with zirconia crowns, indicating favorable material performance under standardized laboratory conditions. These outcomes may be attributed to the controlled digital workflow and homogeneous material structure of CAD-CAM fabricated restorations. The stability observed in surface roughness, microhardness, and color parameters after thermocycling further suggests resistance to simulated aging conditions. Although several differences between groups were statistically significant, these findings are based on in-vitro conditions and should be interpreted with caution. The results indicate potential clinical relevance but do not directly translate to clinical performance or long-term outcomes.

The present study demonstrates several methodological strengths that enhance the reliability and relevance of the findings. A standardized tooth preparation protocol performed by a single calibrated operator minimized procedural variability and ensured consistency across all specimens. The use of a fully digital CAD-CAM workflow further improved precision and reproducibility in crown fabrication. Internal adaptation was assessed using a combination of the silicone replica technique and three-dimensional digital analysis, providing a more comprehensive and accurate evaluation compared with conventional methods. Additionally, the inclusion of both pre- and post-thermocycling assessments allowed for the evaluation of material behavior under simulated aging conditions, thereby offering insights into short-term durability. A key strength of this investigation lies in its comprehensive approach, integrating marginal fit, internal adaptation, mechanical properties, and optical characteristics within a single study design, which remains limited in existing literature. From a clinical perspective, the study holds particular relevance in pediatric dentistry, where full-coverage restorations are frequently indicated for extensively decayed primary teeth. The evaluation of alternative materials such as graphene-reinforced CAD-CAM crowns is important in addressing current clinical challenges related to durability, esthetics, and minimally invasive workflows in young patients. By combining multiple performance parameters with a standardized and reproducible methodology, the present study contributes valuable baseline evidence that may support future translational and clinical research in pediatric restorative dentistry.

This study's limitations should be acknowledged. The in vitro design, despite rigorous standardization, does not fully replicate the complex oral environment, including salivary chemistry, enzymatic activity, thermal fluctuations, and cyclic masticatory loading over time. The use of typodont teeth and metal dies, although advantageous for standardization and reproducibility, differs from the biomechanical behavior of natural teeth and supporting structures, which may influence stress distribution and adaptation outcomes. In addition, crown seating was performed under controlled manual pressure; however, the absence of quantitatively standardized seating force may introduce minor variability in marginal and internal adaptation measurements. While the sample size was adequate to detect statistically significant differences, larger sample sizes in future studies would improve generalizability and statistical power. The study did not include cyclic loading or fatigue testing, which are critical for simulating long-term functional performance, nor did it assess wear characteristics or interactions with opposing dentition. Furthermore, standardization of final crown thickness was not explicitly controlled, which may influence mechanical behavior and fracture resistance. Failure mode analysis was not performed, limiting detailed characterization of fracture patterns and their potential clinical implications. Additionally, the inclusion of multiple outcome measures may increase the risk of type I error; however, this was addressed by defining a primary outcome variable and interpreting secondary outcomes with consideration of multiple comparisons. Future studies incorporating long-term aging protocols, fatigue analysis, and more clinically representative conditions are recommended to further validate these findings.

Despite these limitations, the convergence of excellent marginal fit, improved internal adaptation, superior fracture resistance, and stable surface and optical characteristics positions GCAM crowns as a promising esthetic option for pediatric full-coverage restorations. The digital fabrication workflow facilitates not only precision but also customization, efficiency, and reproducibility — attributes that are highly desirable in contemporary pediatric prosthodontics. However, these in-vitro findings require validation in clinical settings, as the oral environment introduces variables such as cyclic masticatory loading, thermal flux, salivary lubrication, and bacterial biofilms that laboratory simulations cannot fully capture. Future longitudinal clinical trials assessing survival rates, wear behavior, patient and parent satisfaction, and biological outcomes will be pivotal in establishing the definitive role of graphene-reinforced CAD/CAM crowns in pediatric restorative practice.

## 5 Conclusion

Restoration of pulpally treated primary molars remains a clinical challenge, with esthetic demands prompting alternatives to prefabricated zirconia crowns. In this study, graphene-reinforced CAD/CAM (G-CAM) crowns demonstrated superior marginal fit and internal adaptation, as well as higher fracture resistance, compared with prefabricated zirconia crowns, while surface roughness, hardness, and color stability remained comparable before and after thermocycling. These results highlight the potential of G-CAM crowns as a durable, esthetic, and precision-fitted solution for full-coverage pediatric restorations. Future investigations should focus on long-term clinical performance, wear resistance, fatigue behavior, and patient-centered outcomes to establish their definitive role in pediatric dentistry.

## Patient consent

Not applicable.

## Institutional review board statement

Institutional Ethical Committee Review Board (Approval No. SRB/SDC/UG-2116/25/PEDO/135).

## Clinical trial registry number

Not applicable.

## Strengths and limitations

This study demonstrated several strengths, including standardized tooth preparation performed by a single operator, the use of digital superimposition for accurate evaluation of internal adaptation, and the assessment of multiple mechanical and optical properties under simulated aging conditions through thermocycling. However, it could not fully replicate the complex intraoral environment, including saliva, occlusal dynamics, and biological responses, therefore, further long-term in vivo studies are recommended.

## Author contribution

RK performed the experiments, data curation, formal analysis, and prepared figures. PJ conceptualized the study and drafted the manuscript. MS supervised the project, validated the findings, provided resources, and contributed to review and editing. All authors read and approved the final manuscript.

## Data availability declaration

The datasets generated during the current study are not publicly available due to ethical and confidentiality restrictions but are available from the corresponding author upon reasonable request.

## Human and animal statement

Not applicable.

## Sources of funding

Nil.

## Declaration of competing interest

The authors declare that they have no known competing financial interests or personal relationships that could have appeared to influence the work reported in this paper.
